# Multilevel Factors Impacting U.S. School Nurses’ Support of LGBTQ+ Youth: A Qualitative Analysis

**DOI:** 10.1177/10598405251351297

**Published:** 2025-07-04

**Authors:** April J. Ancheta, Alora N. Del Duca, Dominique G. Ruggieri, Catherine C. McDonald, Dalmacio Dennis Flores, Nadia L. Dowshen

**Affiliations:** 1 Perelman School of Medicine, 6572 University of Pennsylvania, Philadelphia, PA, USA; 2 Craig-Dalsimer Division of Adolescent Medicine, Department of Paediatrics, 6567 Children's Hospital of Philadelphia, Philadelphia, PA, USA; 3 PolicyLab, Children's Hospital of Philadelphia, Philadelphia, PA, USA; 4 School of Nursing, 6572 University of Pennsylvania, Philadelphia, PA, USA

**Keywords:** school nurse knowledge/perceptions/self-efficacy, LGBTQ, policies/procedures, qualitative research, school nurse

## Abstract

School nurses (SNs) can play important roles in supporting LGBTQ+ youth. Prior research has emphasized individual and interpersonal factors influencing SNs’ interactions with LGBTQ+ youth, showing mixed results regarding cultural competency, knowledge, and youth satisfaction. However, higher-level factors (e.g., structural and political) shaping these interactions remain underexplored in the United States at a national level. To address this gap, we analyzed open-ended responses from a survey distributed by the National Association of School Nurses in May 2023, receiving responses from 672 nurses (49 states and one U.S. territory). Analysis revealed five key themes: (1) personal stake and the SN role; (2) perspectives on school and community climate; (3) policies as supportive versus challenging; (4) LGBTQ+ resources and education; and (5) barriers and facilitators to learning. Findings illustrate the interconnectedness of policy, leadership, school culture, and education, highlighting areas for targeted improvements to enhance SNs’ capacity to effectively support LGBTQ+ youth health.

Lesbian, gay, bisexual, transgender, and queer/questioning (LGBTQ+) youth are a diverse group of individuals whose gender and/or sexual identity/expression subvert predominant cisgendered and heterosexual societal views and values. This population faces pervasive stigma stemming from cisheteronormativity (i.e., the notion that being heterosexual and/or cisgender are the norm, garnering privilege over other identities) (Marchia & Sommer, 2019). LGBTQ+ stigma informs unjust power dynamics, social oppression, and structural marginalization (Butler, 1973; Foucault, 1978; Rubin, 2002) and influences disparate health outcomes for LGBTQ+ individuals (Franco-Rocha et al., 2023). For example, at the individual level, LGBTQ+ youth experience higher rates of suicide, depression, and anxiety (Hafeez et al., 2017; Johns et al., 2020; Kosciw et al., 2020). At group and societal levels, discriminatory views of LGBTQ+ youth result in unguaranteed family and community support; higher unemployment rates; insecure housing; and insufficient healthcare access and utilization (Henderson et al., 2022). At the policy level, more than 600 legislative bills undermining LGBTQ+ rights were proposed in the United States in 2023 (Trans Legislation Tracker, 2023). These policies included attacks on gender-affirming care and the eradication of LGBTQ+ education and visibility in schools (Movement Advancement Project, 2022; Truong et al., 2022).

K–12 schools are institutions that mirror the power relations of their sociocultural context and actively (re)produce them (Kosciw et al., 2020; Shattuck et al., 2021). Cisheteronormative values influence hierarchical power structures, school climate, and LGBTQ+ school experiences (Kuhlemeier et al., 2021; Shattuck et al., 2021). Aspects such as bullying, dress code policies, LGBTQ+ (in)visibility, and social value signaling cause harm to LGBTQ+ youth (Kuhlemeier et al., 2021). Interactions between community members, parents, staff, and students further contribute to school climate. Their biases, narratives, and misconceptions about LGBTQ+ individuals can interfere with necessary support and positive experiences for these youth (Kuhlemeier et al., 2021; Mann et al., 2024).

As wellness educators, healthcare providers, and public health advocates, school nurses (SNs) can play significant roles in supporting LGBTQ+ youth (Willgerodt et al., 2018). Previous literature demonstrates that LGBTQ+ youth may seek SN assistance at higher rates than their cisgender, heterosexual peers (Rider et al., 2018). For many, SNs serve as key points of access to mental health support, and for some LGBTQ+ youth, the SN may be their only available source of mental health-related assistance (Bohnenkamp, 2015; Bohnenkamp et al., 2019; Maughan et al., 2018). SN-specific services for LGBTQ+ youth often include safer bathrooms (Rider et al., 2018), affirming environments (Bouck, 2020), and LGBTQ+ relevant sexual health education. However, SNs may not feel optimally equipped to serve the unique needs of LGBTQ+ youth. Previous studies that have focused on individual and interpersonal factors influencing SN interactions with these youth have revealed inconsistencies regarding SNs’ cultural competency and cultural humility (Carabez et al., 2015), as well as lack of LGBTQ+ knowledge among SNs (Aisner et al., 2020; Ancheta et al., 2025; Margolies & Brown, 2019). Further, LGBTQ+ youth have reported mixed satisfaction with SN encounters (Laiti, Pakarinen et al., 2024; Laiti, Parisod et al., 2021). LGBTQ+ youth have expressed discomfort speaking with SNs about their gender identities because they felt SNs did not understand or support them. LGBTQ+ youth have also reported SNs’ assumptions of cisgender and heterosexual identities, which contributes to diminished trust and lack of open conversations (Laiti et al., 2021).

Although previous research highlights some of the challenges and opportunities for SNs in addressing the needs of LGBTQ+ youth, the exact position of SNs remains underexplored. The role of the SN warrants further investigation as a potential conduit for combatting the cisheteronormative cultures and policies influencing school environments. Indeed, a critical and timely gap around the interplay of multiple different factors on the capacity of SNs across the United States to support LGBTQ+ youth remains.

Therefore, our study aimed to better understand how multilevel factors (e.g., individual, structural, and political) influence SNs’ experiences and opportunities to better serve LGBTQ+ youth. In so doing, we expand on previous work focused solely on individual and interpersonal levels, as well as barriers that may impact SN practices in singular localities (Shattuck et al., 2025). Ultimately, our research serves as an important steppingstone for recommendations and policies aimed at supporting SNs and ultimately better serving the unique needs of LGBTQ+ youth in K–12 schools.

## Methods

### Study Design and Sample

To capture diverse SN perspectives on the topic of caring for LGBTQ+ youth, our team designed a cross-sectional, national survey in consultation with relevant adolescent medicine, LGBTQ+ youth, health disparities, and psychometrics experts. In conceptualizing our survey, we were informed by the NASN School Nursing Practice Framework that states that school nurses should champion health and academic equity and provide culturally sensitive, inclusive, and holistic care (National Association of School Nurses, 2024). We sought specifically to assess the knowledge and attitudes of school nurses in addressing the needs of the marginalized population of LGBTQ+ youth. We distributed our survey to active members of the National Association of School Nurses (NASN) who opted into a listserv to receive research study participation opportunities. The survey asked SNs about their perspectives on topics such as knowledge of LGBTQ+ youth health issues, attitudes toward LGBTQ+ topics pertaining to data collection and continuing education, and specific SN practices that support LGBTQ+ youth. We also asked five open-ended, optional questions to explore policy and educational experiences surrounding care for LGBTQ+ youth. These questions were: (1) if you would like, please describe your experiences with SCHOOL DISTRICT-WIDE policies and caring for LGBTQ youth; (2) if you would like, please describe your experiences with STATE-WIDE policies and caring for LGBTQ youth; (3) please describe one or more BARRIERS to your learning about LGBTQ youth health needs; (4) please describe one or more FACILITATORS to your learning about LGBTQ youth health needs; and (5) What else you would like us to know?

We emailed a REDCap survey link to a random sample of 2,000 SNs from May to June 2023. Inclusion criteria included actively practicing as an SN in the United States and its territories. Exclusion criteria included SNs who were retired or who held mainly teaching or administrative positions (e.g., SN manager or consultant). Two follow-up email reminders were sent. The study was deemed exempt from full board review by the Children's Hospital of Philadelphia Institutional Review Board (IRB 22-020439).

### Data Analysis

Our team separately analyzed close-ended survey questions pertaining to knowledge and attitudes of SNs in caring for LGBTQ+ youth, as detailed in a separate article (Ancheta et al., 2025). The current analysis focused on qualitative data from the five open-ended questions. We were guided by the Socioecological Model, which highlights how individual, relational, school, community, and policy-level factors can interact to shape SNs’ capacity to support LGBTQ+ youth (Bronfenbrenner, 1979). All responses were exported to Microsoft Excel and data were cleaned to exclude nonresponses, such as blank fields and responses including “none” and “N/A.” Two researchers (AJA and AD) open-coded the data under the guidance of a team member with expertise in qualitative methods (DGR). Following the Braun and Clarke method for thematic analysis (Braun & Clarke, 2006), we separately coded each of the responses to all five questions and subsequently created a codebook that was revised and calibrated through comparison, refinement, and consolidation of the first 30 coded responses for each question. The coders met weekly to settle coding discrepancies from the finalized codebook. After familiarizing ourselves with the data, we then performed a thematic analysis to categorize, frame, and interpret the data, revealing meaningful structural and policy recommendations to support SNs.

## Results

A total of 1189 SNs responded to the survey (59.5% of 2000), and 672 SNs (56.5%) included a free-text response to any of the five open-ended questions. Participants who answered any number of the five questions were included in this study. Table 1 contains participant demographic information for those who completed the open-ended questions. Most of the sample was between ages 50 and 59 years old (35.1%), and identified as White (87.9%), non-Hispanic (96%), female (98.2%), heterosexual (95.1%), and cisgender (99.1%). Most SNs (37.1%) had practiced for 11+ years as a school nurse, 54% worked in pre-K–8th grade, 92% worked in public schools, and SNs were spread from across the country (30% from the south, 26% Midwest, 26% northeast, and 17% from the west). Compared to the full sample, this study sample was older, held more moderate-to-liberal views, received more past-year professional development, and were less from the South (not shown). Table 2 lists the five open-ended questions with frequency of respondents for each question. From our thematic analysis, five central themes emerged: (1) personal stake and the role of the school nurse, (2) perspectives on school and community climate and culture, (3) policies as supportive versus challenging, (4) LGBTQ+ resources and education for students and staff, and (5) barriers and facilitators to learning more about LGBTQ+ youth. These themes are described in detail in Table 3. Table 4 provides additional quotes by theme.

**Table 1.** Participant Demographics.

Variable	*n* (%)
Age	
20–29 years	19 (2.8)
30–39 years	102 (15.2)
40–49 years	188 (28.0)
50–59 years	236 (35.1)
60–69 years	119 (17.7)
70+ years	8 (1.2)
Race	
American Indian or Alaska Native	3 (0.4)
Asian	6 (0.9)
Black or African American	30 (4.5)
White	591 (87.9)
Some other race	16 (2.4)
Multiple races	26 (3.9)
Ethnicity	
Non-Hispanic	645 (96.0)
Hispanic	27 (4.0)
Sex	
Female	660 (98.2)
Male	5 (0.7)
Don't know/prefer not to answer	7 (1.0)
Sexual identity	
Heterosexual	639 (95.1)
Sexual minority	33 (4.9)
Gender identity	
Cisgender	666 (99.1)
Transgender or gender diverse	6 (0.9)
Education level	
Associate's degree	91 (13.5)
Bachelor's degree	378 (56.2)
Master's degree	190 (28.3)
Doctorate degree	13 (1.9)
Religious importance	
Very important	289 (43.0)
Somewhat important	167 (24.9)
Not too important	89 (13.2)
Not at all important	97 (14.4)
Prefer not to answer	30 (4.5)
Political affiliation	
Very conservative	21 (3.1)
Conservative	131 (19.5)
Moderate	225 (33.5)
Liberal	169 (25.1)
Very liberal	60 (8.9)
Prefer not to answer	66 (9.8)
Past-year LGBTQ+ continuing education	
0 h	237 (35.3)
1–3 h	281 (41.8)
4–6 h	72 (10.7)
7+ hours	18 (2.7)
Not sure/do not remember	64 (9.5)
Nursing school LGBTQ+ education	
0 h	456 (67.9)
1–3 h	69 (10.3)
4–6 h	9 (1.3)
7+ hours	11 (1.6)
Not sure/do not remember	127 (18.9)
Total years of nursing practice	
<3 years	20 (3.0)
3–5 years	33 (4.9)
6–10 years	76 (11.3)
11+ years	543 (80.8)
Total years of school nursing practice	
<3 years	127 (18.9)
3–5 years	125 (18.6)
6–10 years	171 (25.4)
11+ years	249 (37.1)
Years at current school nursing employer	
<3 years	169 (25.1)
3–5 years	146 (21.7)
6–10 years	169 (25.1)
11+ years	188 (28.0)
LGBTQ+ family member	
No	237 (35.3)
Yes	394 (58.6)
Not sure	41 (6.1)
LGBTQ+ friend	
No	126 (18.8)
Yes	523 (77.8)
Not sure	23 (3.4)
LGBTQ+ colleague	
No	63 (9.4)
Yes	465 (69.2)
Not sure	144 (21.4)
School type	
Public (including charter schools)	618 (92)
Private (nonreligious)	32 (4.8)
Private (religious)	22 (3.3)
Primary work setting	
Pre-K–8	363 (54)
Middle school	129 (19)
High School	180 (27)
U.S. region	
Northeast	177 (26)
Midwest	178 (26)
South	199 (30)
West	116 (17)

**Table 2.** Open-Ended Questions and Frequency of Responses.

Question	Frequency, *n* (%)
If you would like, please describe your experiences with SCHOOL DISTRICT-WIDE policies and caring for LGBTQ youth	310 (26)
If you would like, please describe your experiences with STATE-WIDE policies and caring for LGBTQ youth	250 (21)
Please describe one or more BARRIERS to your learning about LGBTQ youth health needs	576 (48)
Please describe one or more FACILITATORS to your learning about LGBTQ youth health needs	501 (42)
What else you would like us to know?	307 (26)

**Table 3.** Description of Themes.

Theme	Description
1. Personal Stake and the Role of the School Nurse	SNs expressed motivations and beliefs in supporting LGBTQ+ youth, influenced by personal experiences and professional roles. They emphasized equality and nondiscrimination, despite some conflicting attitudes. Challenges included navigating school policies and community opinions, but SNs relied on their personal beliefs to treat all students with dignity and respect
2. Perspectives on School and Community Climate and Culture	SNs noted their influence on school climate through welcoming policies and nonjudgmental spaces, but acknowledged larger school climate aspects are shaped by students, staff, administration, and parents. They faced challenges like parental opposition to certain curricula and inaction on LGBTQ+ issues by some staff. Community- and state-level attitudes, especially in conservative areas, also impacted schools’ approaches to LGBTQ+ matters. Despite societal progress, traditional beliefs among some SNs and external conservative pressures hindered full acceptance and support for LGBTQ+ youth
3. Policies as Supportive vs. Challenging	SNs highlighted the significant impact of policies on the treatment of LGBTQ+ students, with policies being either supportive or challenging. Leadership and decision making were critical in shaping educational content and practices. While efforts to increase LGBTQ+ inclusion were noted, their implementation depended on district leaderships’ values. Some schools had inclusive policies in general but lacked specific LGBTQ+ protections. SNs also expressed concerns over state and local policies that negatively impact LGBTQ+ youth, with opinions ranging from strong opposition to confusion or lack of information.
4. LGBTQ Resources and Education for Students and Staff	SNs emphasized the need for inclusive and accessible staff training on LGBTQ+ matters, with effective in-person sessions during school hours. Some SNs provided specific resources for LGBTQ+ students, such as safe spaces and support for mental health concerns. Despite restrictive policies, some SNs continued to use students’ preferred names and pronouns. Challenges included outdated systems for recording gender identity and a need for better support from schools in training on LGBTQ+ issues. For students, they noted that puberty and sexual health education for students is often watered down
5. Barriers and Facilitators to Learning More About LGBTQ+ Youth	SNs identified time constraints and a lack of tailored learning opportunities as major barriers to learning about LGBTQ+ youth. Community- and state-level conservatism also hindered education efforts. Facilitators included in-school trainings, support from other staff, online forums, local LGBTQ+ organizations, and professional nursing associations. Personal willingness, professional duty, and external pressures also encouraged SNs to seek more knowledge about LGBTQ+ issues

LGBTQ= Lesbian, gay, bisexual, transgender, and queer/questioning; SN=school nurse.

**Table 4.** Additional Quotes by Theme.

*Theme 1—Personal Stake and the Role of the School Nurse*	
Quote 1	“I have learned that being neutral, not doing or saying anything to support LGBTQ students is the same as taking the side of the oppressor. I agree with that statement and don't agree when the district is silent. I was also silent but have begun to be more openly supportive of students”
Quote 2	“I know some religious beliefs in the state of Utah are against LGBTQ. My own religious beliefs involve the belief that marriage is between a man and woman. Procreation can only naturally happen this way. That being said, my religion also teaches that ‘Jesus said love everyone, treat them kindly too.’ I may not love choices people make, but I can still love the people. We are all just trying to figure life out. So, I know the Mormons get a bad rap for funning the state of Utah religiously with judgement. I feel that is changing in Utah. I should pay more attention to the LGBTQ platform to ensure all humans are loved and created equal, no matter what their orientation or personal beliefs are”
Quote 3	“The movement to increase teaching in these areas is confusing to children and we are seeing a tremendous increase in mental health problems secondary to children being confused about something that is fundamental to biology”
Quote 4	“The answer to any unknown in out district at HS level is ‘send to the nurse’ or ‘use nurse bathroom’. We have more lee-way than some other staff roles b/c we can address and assess based on the medical problem/symptoms—that means we MUST ask questions about biological sex, puberty blocking or transitional treatments such as hormones, etc that might cause symptoms, we can also ask about sex-safety measures to help us refer appropriately to further care”
Quote 5	“In my school and in my life, I treat everyone with the kindness and care that they deserve. Sexual orientation has no factor in the care they receive”
*Theme 2—Perspectives on School and Community Climate and Culture*	
Quote 1	“This district is very open and continues to advocate for LGBTQ+ youth”
Quote 2	“Despite a conservative school board that can create barriers, there is strong support in the district's administration and leadership that advocates for equity, diversity, and inclusion in the school setting”
Quote 3	“The kids that do identify as LGBTQ are too scared to say anything and those that don’t tend to bully those that do. It is a very hostile environment when it comes to LGBTQ issues”
Quote 4	“School board and attitude of the community make it difficult to implement research backed health initiatives/procedure changes, and in my opinion this greatly restricts what the district can implement to support LGBTQ students”
Quote 5	“School district is very conservative Christian, so LGBTQ youth are seen as needing help to regain their assigned birth gender identity and desire for heterosexual lifestyle. Of course we have LGBTQ students, but they can’t come out at school”
*Theme 3—Policies as Supportive vs. Challenging*	
Quote 1	“The school district has not been able to provide us with their policy & procedure manual”
Quote 2	“I would like to be able to hang a poster/picture in my office identifying the space as a ‘safe space’ for students that are LGBTQ. But posters like that are against board policy”
Quote 3	“I care for all children with no judgment based on the decisions they make. I do not need a policy to enable me to give good care”
Quote 4	“State-wide policies and caring for LGBTQ youth has been constricting for that population of students. It can be perceived as a means of elimination or isolating students because of their gender identity”
Quote 5	“I haven't had any personal experience with the state wide policies but I have heard many say that if they have to teach students about any LGBTQIA+ they will resign first”
*Theme 4—LGBTQ*+ *Resources and Education for Students and Staff*	
Quote 1	“We need MORE support and more training on how to accomplish that support!!!”
Quote 2	“Our school district provides one required course on this topic and then it is never spoken about the rest of the year”
Quote 3	“Knowing the best and most reputable places to look for evidence-based and up to date help!”
Quote 4	“Our school is mandated to provide sex education/family life classes. These classes are dumbed down in my opinion. Many years ago we taught about sexual education but now we only teach on basic hygiene. There are a few anatomy things we touch on but the curriculum is very watered down. These students hear so much misinformation at school and many have no one to ask/clarify what is real”
Quote 5	“I would love to have some sort of program that is virtual that could provide a certification in LGBTQ resources and training, especially geared to the school nurse. School nurses do so much mental health care in our schools, yet we don’t have the proper training”
*Theme 5—Barriers and Facilitators to Learning More About LGBTQ*+ *Youth*	
Quote 1	“The main facilitator to my learning about LGBTQ youth health needs is speaking directly to the student”
Quote 2	“One of my sons is gay and the other is pan. I actively look for ways to support them and my students through our school counselors and through social media and LGBTQ groups”
Quote 3	“Rely upon own resources to find evidence based educational offerings on subject, along with time constraints”
Quote 4	“I have no barriers to learning. However, I have no desire”
Quote 5	“Opportunities are few but I do not seek them out”

LGBTQ= Lesbian, gay, bisexual, transgender, and queer/questioning.

### Theme 1: Personal Stake and the Role of the School Nurse

School nurses articulated varying motivations for supporting LGBTQ+ youth, which included a mixture of personal beliefs; experiences; and the inherent professional roles they perceive themselves playing within healthcare and educational settings. While some SNs expressed a strong, positive alignment with equality and inclusion, others revealed conflicting or negative attitudes that stemmed from personal views.

One SN noted:

*I do not put sexual identity in the minds of elementary age children. I refuse to take away the parents role. These topics are between the students and their parents. Not for Government.*

*—Pre-K–8 SN from Texas*

Another SN said:

*When I began to understand the stigma that is attached to being LGBTQ, I was horrified. I then became much more empathetic and opened my heart to understand what these individuals deal with on a daily basis. I am proud to say that I love working with students who identify as LGBTQ and I consider myself to be one of their strongest advocates now.*

*—Middle school SN from Massachusetts*

Despite varying individual-level views, a notable point of consensus among SNs emphasized the importance of providing respect and dignity to all students. Many nurses cited this as a key motivator for adopting inclusive practices (e.g., physically identifying their health office as safe spaces or respecting students’ identity wishes), which aligned with their professional commitment to being a nondiscriminatory provider.

*In my school we use the health office bathroom if a student wants to use the bathroom of their chosen gender. My school is only 6th graders. We are seeing more students that are open about their gender and LGBTQ health needs. We try to treat all students with caring and respect.*

*—Middle school SN from New Jersey*

The aspiration to be an inclusive LGBTQ+ provider was noted as sometimes challenging, characterized by tension and frustration when SNs’ personal stakes, nursing values, and ability to support LGBTQ+ youth conflicted with external factors such as administrative directives or community or parental opinions.

*In my district we are not supposed to offer any sexual health care, we are supposed to preach abstinence only which is detrimental… That said I do a lot of other things, I just have to watch my back when doing so. I have argued that my duty as a nurse and health care provider with my patients comes first and district policy comes way down my priority list.*

*—High school SN from North Carolina*

From this quote we can hear the SN's balance between wanting to be an inclusive provider and the directive sent down from administration. Contention with personal beliefs and typical duties expected of SNs by their employers were highlighted by several SNs.

### Theme 2: Perspectives on School and Community Climate and Culture

SNs commented on the influence of their practices and care on the overall school climate. Specifically, welcoming open-door policies and nonjudgmental spaces contribute to inclusion and belonging. However, SNs noted that many aspects of the school and political climate locally and/or nationally had restrictive influence on the education or support they could provide for LGBTQ+ youth. This impacted, for example, their ability to provide puberty and sex education; to fully affirm students’ pronouns, sexual orientation, and/or gender identity; and to sufficiently address physical and mental health needs that are particularly salient for LGBTQ+ youth.

One SN comments:

*I work for a religious private school. They do not acknowledge any LGBTQ descriptors. I have encountered 4 students in my practice who have confided in me their preferred pronouns, sexual orientation and/or gender identity. Unfortunately where I work, there is too much stigma & resistance.*

*—Pre-K–8 SN from Wisconsin*

Another SN connects the community climate with possible outcomes for LGBTQ+ students:

*I feel as though living in a conservative leaning state, resources and care for LGBTQ youth is in jeopardy…[and there are] barriers towards the healthcare and mental health needs of our LGBTQ youth…putting these youth in much higher danger for bullying, harassment, mental health concerns, and risk for suicide.*

*—Pre-K–8 SN from Wisconsin*

Many SNs explained that schools in rural or conservative environments do not discuss LGBTQ+ matters due to local community norms, with some noting that negative rhetoric espoused by state legislators can affect how they practice in their schools. On the other hand, there were SNs who explained more supportive school climates and how they were encouraged from a policy and systems-level perspective.

*Our educational service district has very strong policies and mission statements encouraging and mandating equitable education access, treatments, personal supports and safe areas for all students (and staff) including members of LGBTQ+, BIPOC, Physical/Mental Health and neuro-diverse communities.*

*—High school SN from Oregon*

This SN not only comments on the inclusivity of students by sexual orientation and gender identity, but also by other marginalized identities like race/ethnicity and neurodiversity.

### Theme 3: Policies as Supportive Versus Challenging

In addition to noting their personal stake and the influences of school and community climate, SNs also described the dichotomy of policies as supportive versus challenging for LGBTQ+ students and themselves. Leadership, power, and decision making were highlighted as critical elements for shaping educational content and school practices, with additional complications stemming from the rapidly changing nature of school environments and student demographics.

One SN comments on how the nonexistence of a curriculum and related policy influenced their decision to not continue doing the typical education they would be doing:

*I am not doing any type of puberty training in my elementary school this year due to the crazy current political climate. I am saddened by this, but unless I have the backing of a written, spelled out curriculum from the district for puberty and sex education, I will no longer be doing this.*

*—Pre-K–8 SN from Ohio*

SNs sometimes encountered LGBTQ+ supportive policies related to discrimination and bullying, deeming these policies as supportive. However, SNs noted decreased effectiveness of implementation due to a lack of comprehensive understanding of policies and lack of concrete support for enforcement.

*The policies are in place but not always followed in the schools.*

*— Pre-K–8 SN from Vermont*

However, some SNs did explain when policies worked for LGBTQ+ students. This SN specifically reflected on how their state school nurse organization hired lobbyists to support such policies:

*Through development of legislative priorities our state school nurse organization promotes legislation for these policies that protect LGBTQ students of all ages and have hired lobbyists engaged in this effort.*

*— Pre-K–8 SN from Massachusetts*

Overall, SNs expressed mixed feelings over policies that directly target and negatively impact LGBTQ+ youth. These include regulations that restrict gender-affirming care, exclude transgender athletes from sports, and impede inclusive education, among others. SNs’ opinions on these matters ranged from strongly opposed to supportive, with other comments revealing confusion or feelings of being uninformed.

This one SN notes the effects of a school-level policy that they would be involved with for an LGBTQ+ student:

*There is current legislation on the table that would require the school to notify parents within 5 school days if a student requests to be called by a different name. I am afraid for my students.*

*—High school SN from Indiana*

### Theme 4: LGBTQ+ Resources and Education for Students and Staff

Many nurses commented on the different ways through which SNs, staff, and students learn about LGBTQ+ health and wellbeing. Some of the most successful trainings noted by SNs were those conducted in-person during school hours with space for asking questions openly and anonymously. SNs expressed that trainings should be welcome to all learner levels. One SN noted:

*Sometimes I'm shamed by adults in the school who are super pro LGBTQ … if I accidently misgender a student or try to ask a legitimate question. I want to learn about a culture that has been in the shadows for years and believe you do better when you know better. Nobody wants to feel scolded when you are trying to get better.*

*—High school SN from New York*

This theme also encompasses SN-specific resources offered to LGBTQ+ students (e.g., a safe, nonjudgmental space with an advocate to whom they can confide).

*My office provides a safe space for LGBTQ students. My office provides a restroom, a changing room for p.e. or a room just to chill out.*

*—High school SN from Illinois*

SNs also noted the help of other staff in their learning:

*Our counselors and other support staff have signs on their doors that provide a visual that their office is a safe space for these students. I recognize that I need one as well.*

*—Pre-K–8 SN from Delaware*

SNs discussed electronic medical records systems, commenting on outdated aspects of identity documentation (e.g., the sex field including only options for male/female). But they also reflected on the importance of medical records as an aid to recall preferred name and pronouns if their system allowed such documentation.

One SN notes how the medical records system plays a role in their school:

*We now have the ability to change the student's name and preferred gender in the student record system. However, for medication- the legal name must be given on the orders.*

*—Pre-K–8 SN from Washington*

For the student resources topic, many SNs commented on puberty or growth and development classes for elementary-level students and sexual health education for middle and high school-level students. These were core classes available at the K–12 level through which students could learn about sexual orientation and gender identity and the unique health needs of this population.

### Theme 5: Barriers and Facilitators to Learning More About LGBTQ+ Youth

The majority of SNs stated that time was a primary barrier for them learning about LGBTQ+ matters—time to take continuing education, prioritize this type of learning over other subjects, and time on the job for trainings given their demanding workflow.

*Time. LGBTQ is only one issue amongst many mental, social, and physical well being issues that need to be adequately addressed with continuing education*

*—Pre-K–8 SN from Virginia*

A significant lack of learning opportunities was another major barrier. SNs discussed that trainings offered by schools/organizations focused on generalized education about LGBTQ+ topics or were targeted to teachers rather than to SN-specific issues. SNs also mentioned that access to free, up-to-date, and evidence-based trainings offered in-person during school hours are most useful. Some SNs stated that trainings specific to their local and/or religious communities would be helpful.

This SN comments on the importance of trainings being developed by school nurses for school nurses, as well as the barrier of time:

*Truthfully not having enough time, and not having access to individuals that understand what school nurses need to know. I've attended various conferences and professional development but not developed for school nurses by school nurse to address issues specific to school nursing.*

*—Pre-K–8 SN from California*

Despite these barriers, SNs noted some facilitators to their learning more about LGBTQ+ student needs. Major educational sources included all-learning level trainings, other staff such as counselors and social workers, forums, local LGBTQ+ organizations, and SN organizations.

*We have open communication between students, parents, administrators, nurses, guidance counselors, and other school staff to communicate the needs of LGBTQ students and to meet those needs so those students feel school connectedness and are comfortable.*

*—Middle school SN from Pennsylvania*

Some SNs noted that an LGBTQ+ educational requirement by state boards of nursing licensing organizations could be most impactful as that would directly impact the need for LGBTQ+ cultural humility training at any entry-to-practice level.

Lastly, SNs mentioned that personal willingness and curiosity; professional duty; and support or pressure from family, friends, or students were also facilitators to learning.

Still, some SNs felt as though there is no need to receive training on students based on their identities. One SN comments:

*I’m not going to treat LGBTQ students any differently than I would their peers. I don’t need specialized learning regarding LGBTQ youth health needs. They have the same needs as any other youth in today's times. Most kids are struggling over something and need help in various ways. As a nurse, I do what I can for the INDIVIDUAL, not their identity.*

*—High school SN from Alabama*

## Discussion

In this article we aimed to uncover the multilevel factors that influenced SNs’ abilities to provide better care for LGBTQ+ students in K–12 United States (U.S.) schools. Through a thematic analysis of open-ended, free-text response data from 656 school nurses, we found five inter-related themes: (1) personal stake and the role of the school nurse, (2) perspectives on school and community climate and culture, (3) policies as supportive versus challenging, (4) LGBTQ+ resources and education for students and staff, and (5) barriers and facilitators to learning more about LGBTQ+ youth. Although the topics of the open-ended questions mainly focused on policy impacts as well as educational barriers and opportunities, SNs offered much more information and insights into their practices, particularly motivations and perceived care roles for LGBTQ+ youth. The high percentage of response frequencies for each question revealed that SNs had a large interest in having their voices heard on this topic. Overall, analysis of SN responses painted a broad picture of the many influences of internal and external forces that affect SNs’ care of LGBTQ+ youth.

Our examination began at the individual level, revealing a conflict between SNs’ self-defined roles as providers, advocates, and educators, and the external factors that challenge these roles. Despite opposition, many SNs remained dedicated to ensuring that LGBTQ+ youth receive the care and support they deserve, highlighting the pivotal role SNs play in fostering inclusive school environments, as supported by previous research (Bergren, 2017; Kvarme et al., 2020; Ladd, 2009). On the interpersonal and organizational levels, SNs observed that school and community climates are shaped by various actors, with state and community rhetoric influencing school rhetoric and practices. This trickle-down effect can result in either support for or erasure of LGBTQ+ students. A 2023 Trevor Project survey found that 86% of transgender and gender-diverse youth reported negative mental health impacts from public discourse on antitrans bills (The Trevor Project, 2023), reinforcing SNs’ observations that school climates are deeply affected by broader policy discussions.

At the structural level, the dual impact of policies revealed that supportive policies help protect and affirm LGBTQ+ students while challenging policies contribute to exclusion and harm. The absence of LGBTQ+-specific policies was a major concern, echoing prior research that inclusive policies improve health outcomes for LGBTQ+ individuals (Krantz et al., 2024; Meyer et al., 2019). Future research should explore factors influencing policy inclusion and exclusion. Education, such as suicide prevention training and gender diversity topics, was seen as a sign of acceptance and inclusivity. Conversely, the lack of such education perpetuated gaps and stigma around LGBTQ+ issues. Barriers and facilitators to SNs’ learning about LGBTQ+ topics spanned all levels of the socioecological model. Influences ranged from individual factors, like religious views and personal curiosity, to structural factors, such as cost barriers and supportive school administration.

Our findings add significantly to the nascent literature on SNs and macrolevel factors that influence their ability to provide best care for LGBTQ+ youth in the United States. Laiti et al. (2024) found similar themes to ours in their qualitative analysis of interviews with junior high SNs on their experiences and perceptions in supporting LGBTQ+ students in Finland. They found four main themes in their work: professional identity and practice, recognition of sexual and gender diversity in school, family acceptance process, and LGBTQ students as school nursing clients. Our theme of personal stake and role of the SN relates to Laiti's findings about a heightened sense of professional duty to care for all students respectfully, regardless of identity. Additionally, Laiti's research similarly highlighted SNs’ recognition (or lack thereof) of LGBTQ+ matters in school and the role of family and parents in influencing what can and should be discussed regarding gender and sexuality in the classroom. SNs in our sample also spoke to personal experiences caring for LGBTQ+ youth, similar to Laiti's theme of LGBTQ+ students as client, but many in our study spoke to never encountering these students due to the schools in which they work (e.g., elementary schools) or specialty schools (e.g., schools that serve students with disabilities). Our study adds more contextual information on the influence of climate and policies on the practice of U.S. SNs.

In a similar 2021 qualitative study based on research from New Mexico, Shattuck et al. (2021) found that implementation of evidence-informed practices to support LGBTQ+ youth was impacted by resource scarcity (e.g., staff turnover), community backlash and opposition to change (e.g., instruction on identity beyond the gender binary), school leadership, and various school and state policies that communicate attitudes about LGBTQ+ students. Cisheteronormativity and its resulting stigma are forces of power that are co-created in institutions like schools. Shattuck stated that power can, “bound, shift, amplify, and otherwise shape the way new practices are received, implemented, and sustained” (Shattuck et al., 2021). Relating to our themes on policies and LGBTQ+ resources and education, higher-level administrators (i.e., school boards and principals) greatly influence resource allocation, responsiveness, and authority. This dynamic molds the environments in which SNs practice, therefore constraining LGBTQ+ support. However, SNs also disrupt cisheteronormativity by providing inclusive spaces that interrogate gendered stereotypes and question traditional structures and organizational norms.

In an analysis of barriers and facilitators to implementing evidence-informed practices to enhance school climate and health outcomes for LGBTQ+ youth, in a separate study, Shattuck et al. (2025) found that time, heavy workload, lack of staffing resources, and staff turnover were all entities that prevented SNs in New Mexico from best supporting the needs of LGBTQ+ students. These results are also very much in line with responses from SNs on barriers in our survey, indicating the similarities that SNs across the country may experience, even given different state geographies and student demographics.

Our work ultimately revealed central themes that impact SN care for LGBTQ+ students in schools with varying structural and political environments across the United States. Despite these differences, all SNs had one thing in common—a strong personal identity as a nurse who cares for children and youth. Prior research has shown that an SN's self-perception as trustworthy and influential in the physical and mental health of youth is related to increased confidence and effectiveness in their roles (Taylor-Beirne & Taylor-Beirne, 2022). With support and encouragement from peers, staff, and students, SN can grow to become competent in these roles (Kaskoun & McCabe, 2022). Cultural humility, an approach that emphasizes ongoing self-reflection, recognition of personal biases, and respect for others’ experiences, should be central to trainings for SNs (Foronda et al., 2016). These trainings can help SNs examine their own identities and assumptions while developing the skills to support the diverse needs of LGBTQ+ youth.

Our findings also have important implications for future research. Future research should seek to incorporate interviews with LGBTQ+ students to capture their firsthand perspectives on school-based support systems, with particular emphasis on exploring the unique experiences of transgender and gender-diverse youth. Additionally, research should investigate best practices for shifting from “safe spaces” to “brave spaces,” which promote more active engagement and resilience-building (Arao & Clemens, 2023). As not every school has an SN, studies should examine the contexts of schools without SNs and the implications for LGBTQ+ youth and other vulnerable groups. Moreover, further inquiry should focus on local-level policy development and implementation to better understand how policies translate into practice and ultimately impact LGBTQ+ student wellbeing. Lastly, future work might also intentionally examine SNs’ perspectives by grade level (e.g., elementary vs. high school), as developmental milestones and understanding of LGBTQ+ issues evolve over time.

### Strengths and Limitations

This study captured diverse perspectives from SNs across 49 states and one U.S. territory. A randomized participant selection approach combined with a 59.5% response rate indicates substantial population interest. Additionally, the anonymous, open-ended questions enabled SNs to authentically express their experiences supporting LGBTQ+ youth, yielding rich, multilevel insights into sociopolitical and structural factors influencing LGBTQ+ care in U.S. schools.

However, our findings lack representativeness to broader LGBTQ+ support in U.S. schools, since we did not include the perspectives of schools without a school nurse and excluded SNs not affiliated with NASN. Participants providing open-ended responses (*n* = 672) were also more likely to identify as moderate–liberal; received more past-year professional development in LGBTQ+ topics compared to the full sample (*n* = 1189); and were more likely to serve pre-K to grade 8 populations, suggesting potential self-selection bias and leading to limited transferability in our findings. Nevertheless, open-ended participants expressed diverse views on LGBTQ+ youth care and education, underscoring the breadth within the sample. Additional limitations include variability in response length, nuance, and relevance, potentially influenced by survey fatigue due to question placement at the survey's end. Lastly, data collection occurred in summer 2023, reflecting the specific sociopolitical context of LGBTQ+ school environments at that time.

## School Nurse Implications

Insights from this study inform critical recommendations for SNs, school administrators, and policymakers. Primarily, the data suggest that SNs require further LGBTQ+ education, resources, and support to bolster their care for LGBTQ+ youth. Our analysis of closed-ended questions from this survey on SN knowledge and attitudes found that past-year professional development on LGBTQ+ topics was associated with both higher knowledge scores and more positive attitudes toward LGBTQ+ students (Ancheta et al., 2025). Effective education for SNs should be free, in-person or virtual, during school hours, sensitive to all learning levels, and relevant to SNs. Appropriate lessons should continue to encourage nondiscriminatory practices in combination with the deconstruction of difference blindness (i.e., the avoidance of recognizing and addressing differences among individuals, particularly in relation to identities) (Smith & Shin, 2014) and its potential harms. Moreover, we encourage SNs to continue establishing safe spaces, while prioritizing student needs, confidentiality, and evidence-based practices. Findings also suggest that strengthened collaboration between SNs and other staff like social workers and guidance counselors improves care coordination for youth and LGBTQ+ knowledge.

With structural, organizational, and political factors within and external to U.S. schools heavily influencing SN practices, school administrators must confront cisheteronormativity and evaluate the extent of LGBTQ+ support in their institutions. The explicit acknowledgement of LGBTQ+ issues, inclusion of justice-oriented school policies and practices, and the uplifting of SN and other staff support roles all comprise an essential foundation to LGBTQ+ student inclusivity. We encourage clear communication and implementation of these support strategies. In addition to explicit language supportive of LGBTQ+ care, protective policies need to extend to SNs and staff themselves, considering possible community and parental opposition.

## Abbreviations

LGBTQ+: lesbian, gay, bisexual, transgender, queer/questioning, and other expanding sexual and gender identities
NASN: National Association of School Nurses
SN: school nurse
